# Magnetically Localized Detection of Amplified DNA Using Biotinylated and Fluorescent Primers and Magnetic Nanoparticles

**DOI:** 10.3390/bios15030195

**Published:** 2025-03-18

**Authors:** Etienne Orsini, Franz Bruckert, Marianne Weidenhaupt, Orphée Cugat, Paul Kauffmann, Sarah Delshadi

**Affiliations:** 1 MagIA Diagnostics, 15 Rue Maréchal Leclerc, 38130 Échirolles, France; paul.kauffmann@magia-diagnostics.com (P.K.); sarah.delshadi@magia-diagnostics.com (S.D.); 2 Univ. Grenoble Alpes, Centre National de la Recherche Scientifique Grenoble Institut National Polytechnique, Laboratoire des Matériaux et du Génie Physique, 38000 Grenoble, Francemarianne.weidenhaupt@grenoble-inp.fr (M.W.); 3 Univ. Grenoble Alpes, Centre National de la Recherche Scientifique, Grenoble Institut National Polytechnique, G2Elab, 38000 Grenoble, France; orphee.cugat@grenoble-inp.fr

**Keywords:** DNA amplification, PCR, LAMP, fluorescence, magnetic nanoparticles, biotin, cyanine 5

## Abstract

Quantitative nucleic acid detection is widely used in molecular diagnostics for infectious diseases. Here, we demonstrate that the previously developed MLFIA (magnetically localized fluorescent immunoassay) has the potential to detect Polymerase Chain Reaction (PCR) and loop-mediated isothermal amplification (LAMP) products using biotinylated and fluorescent primers and streptavidin-coated magnetic nanoparticles. The functionalized nanoparticles separate amplified DNA from non-incorporated primers in situ, allowing the quantification of DNA products. We compare magnetically localized fluorescence detection to commercial technologies based on the DNA intercalation of fluorescent dyes. Our system allows the detection of PCR and LAMP products but is approximately 10 times less sensitive than standard commercial assays. Future optimizations, such as enhancing the signal-to-noise ratio and improving nanoparticle functionalization, could significantly increase sensitivity and bring it closer to current diagnostic standards. This work highlights the potential of magnetically localized fluorescence detection to detect DNA.

## 1. Introduction

Polymerase Chain Reaction (PCR) is a widely used molecular biology technique to amplify and detect nucleic acid sequences [1]. PCR is used for numerous applications across clinical diagnostics, genetic research, forensic science, and environmental monitoring. The power of PCR lies in its ability to amplify small quantities of DNA to high detectable levels, allowing for the precise detection of genetic material. Traditional PCR methods involve multiple steps: sample preparation, DNA extraction, amplification, and detection. Each step is critical and requires careful execution to avoid contamination and ensure accurate results [2]. Despite its efficacy, conventional PCR is often time-consuming and labor-intensive, involving several manual handling steps and generating biological waste that must be disposed of according to strict biosafety protocols. In research laboratories, PCR amplification is performed using a basic thermal cycler, with detection achieved through agarose gel electrophoresis. Quantitative PCR (qPCR) devices have been developed that integrate amplification and real-time detection in a single step, significantly speeding up the process and providing quantitative data [3]. However, these qPCR machines are more expensive than PCR, often requiring substantial investment in equipment and reagents, which may not be feasible for all laboratories.

In clinical diagnostics, qPCR is employed to detect nucleic acids to highlight pathogen infections or genetic mutations. Its high sensitivity and specificity make it an invaluable tool in the early detection and monitoring of diseases, including infectious diseases like SARS-CoV-2 [4] and genetic diseases [5]. Technological advancements have significantly improved qPCR workflows. Automated qPCR systems have been developed to streamline the process, reducing hands-on time and increasing throughput. These systems, such as the Roche Cobas 6800/8800 Systems [6], incorporate automated sample preparation, amplification, and detection, providing rapid and reliable results. They have become indispensable in high-throughput settings like hospitals and large laboratories. Despite these advancements, automated qPCR systems remain expensive and require sophisticated infrastructure and trained personnel, limiting their accessibility in low-resource settings. This has driven the development of portable and user-friendly qPCR devices, allowing users to test people at the point of care. Such devices, including portable qPCR platforms like the BioFire FilmArray [7] and the Cepheid GeneXpert [8], offer rapid and accurate testing capabilities outside traditional laboratory environments. These innovations are crucial for the timely diagnosis and management of diseases, especially in remote and underserved areas. In infectious disease diagnostics, results can also be read using lateral flow assay (LFA) strips [9] instead of agarose gels. LFAs provide a simple, rapid, and visual method for detecting amplified DNA, making them an attractive alternative for obtaining quick results. Recent developments in molecular biology technology focus on further simplifying the process and minimizing the need for specialized equipment. Isothermal amplification techniques, such as loop-mediated isothermal amplification (LAMP), provide a promising alternative to traditional PCR by eliminating the need for thermal cycling [10]. This technique is being exploited by companies such as Eiken Chemicals [11] and Meridian Bioscience [12] for its potential for rapid, high-throughput, and cost-effective testing, making molecular diagnostics more accessible and scalable. Portable and user-friendly qPCR devices, along with alternative techniques such as isothermal amplification, are emerging to make molecular diagnostics more accessible and efficient, particularly in remote and underserved areas. These innovations are crucial for rapid diagnosis and disease management, thereby contributing to the improvement of public health.

MagIA Diagnostics is developing rapid immunoassays based on a technology that combines micro-magnets, magnetic nanoparticles (MNPs), and fluorescence detection. The sample containing the molecule of interest is incubated with functionalized MNPs with specific antibodies or antigens and a fluorescence-detection antibody. After the formation of the immune complexes, the MNPs are locally captured on high-magnetic-field gradients of the micro-magnet array consisting of stripes of alternating magnetization (100 μm large and 10 mm long) at the junction between micro-magnets. The measurement of differential fluorescence between the specific signal at the micro-magnet junction stripes and the background noise between the micro-magnet junction stripes allows for the quantitative detection of the molecule of interest in less than 30 min [13]. Based on this technology, MagIA Diagnostics developed two technologies: the first is a magnetically localized fluorescent immunoassay (MLFIA) dedicated to performing rapid laboratory immunoassays, and the second one is integrated to perform multiplex immunoassays at the point of care [14].

The MLFIA operates as follows [13]: a biological sample containing the target analyte is mixed in a microtube with functionalized MNPs and fluorescently labeled with detection antibodies (Ab), both specific to the analyte. The mixture undergoes incubation for 15 min to facilitate the formation of immune complexes (MNP-target-Detection Ab). Following incubation, the mixture is injected into a microfluidic cartridge that comprises 18 individual chambers, each incorporating a micro-magnetic array forming a stripe pattern. The cartridge is then positioned above a magnetic array known as the MagActivator, which generates a localized magnetic field that facilitates the localized capture of immune complexes on the micro-magnets. The cartridge is subsequently inserted into the analyzer, an optical imaging device specifically designed for fluorescence quantification. The analyzer sequentially captures fluorescence images from each chamber. The fluorescence associated with the captured immune complexes is spatially localized along the micro-magnet stripe patterns, forming distinct fluorescent lines. Fluorescence detected between the micro-magnet lines corresponds to unbound detection antibodies, representing background noise. The image-processing algorithm integrates fluorescence intensities along the micro-magnet lines while subtracting the non-specific background signal, ensuring the accurate quantification of the analyte. The integrated signal is directly proportional to the analyte concentration, providing a quantitative readout.

**Figure 1:**
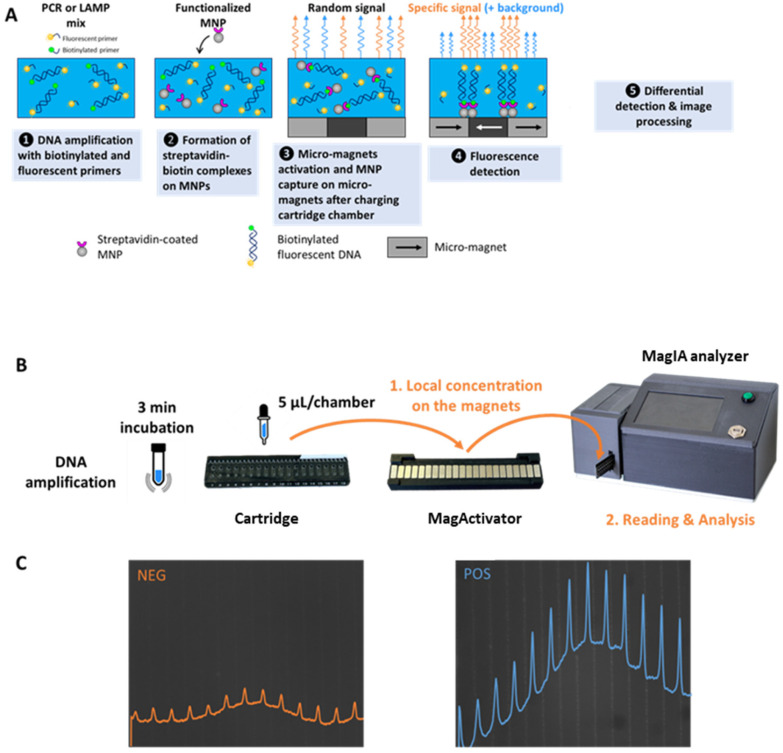
Principle of magnetically localized fluorescence molecular assay (MLFMA) detection of nucleic acids and the experimental workflow. (**A**) Biotinylated and fluorescent amplified DNA molecules are bound by streptavidin-coated magnetic nanoparticles (MNPs) that are captured on the micromagnets after activation by the MagActivator. Due to the strip-shaped micromagnet layout, the magnetically localized DNA molecules form fluorescent lines. (**B**) Workflow and pictures of the cartridges, MagActivator, and MagIA analyzer used to detect MLF signals. (**C**) Pictures with fluorescence profiles. **Right**: negative result; the fluorescence is diffuse. The MLF signal obtained is 19. **Left**: negative result; the fluorescence is on micromagnet lines. The MLF signal obtained is 90.

In this article, we propose exploiting the same magnetic localized fluorescence technology to detect amplified DNA molecules (Figure 1). Throughout this article, we will refer to this new test for detecting amplified nucleic acids as magnetic localized fluorescent molecular assay (MLFMA). To do so, we amplified DNA using primers coupled to biotin and primers coupled to a fluorescent probe (cyanine 5). After the amplification, we incubated the amplified DNA products with MNPs functionalized with streptavidin to capture the fluorescent amplicons. Fluorescent magnetic complexes were then trapped locally in the 18-chamber cartridge. Detection was achieved with the same analyzer previously presented in [13], hereafter designated as the MagIA analyzer. A magnetically localized fluorescence score (MLF signal) is calculated, enabling the quantitative detection of amplified DNA.

Integrating protein and nucleic acid detection in a single, fast, and small platform would facilitate infectious disease management, particularly in laboratories with re-source-limited settings. A new device combining immunoassays and molecular diagnostics would represent a major advancement in the numerous cases where immunoassays and molecular assays need to be performed together (e.g., for diagnosis confirmation or to guide treatment decisions). This work is a novel proof of concept for nucleic acid detection using an already existing immunotesting device, demonstrating its potential for expanded applications in molecular diagnostics.

## 2. Materials and Methods

### 2.1. Magnetic Nanoparticle Functionalization

Carboxylic acid-coated polystyrene magnetic nanoparticles with a 200 nm diameter incorporating a superparamagnetic iron oxide core (M1-020/50) were obtained from Merck GmbH (Darmstadt, Germany). The stock concentration of the MNPs is 1.5 × 10^13^ MNPs·mL^−1^. Streptavidin (189730), ovalbumin (A5503), 1-ethyl-3-(3-dimethylaminopropyl) carbodiimide (EDC) (E6383), MES (M3671), PBS (P4417) and Denhardt solution (D2532) were purchased from Sigma Aldrich (Saint-Louis, MI, USA).

Streptavidin and ovalbumin were prepared at 1 mg·mL^−1^ in 100 mM MES-Na (pH 5.5). Concentrations were checked by UV absorbance at 280 nm. Two-hundred-nanometer carboxylic acid-coated MNPs (1.2 mg, 12 µL, 1.8 × 10^11^ MNPs) were first activated with 100 μL of 1 M EDC in 100 mM MES-Na (pH 5.5) for 30 min at room temperature under agitation. Then, the supernatant was removed, and the MNPs were incubated for 1 h at room temperature with 100 μL of 1 mg·mL^−1^ streptavidin or ovalbumin in MES-Na. MNPs were magnetically captured to remove the supernatant and used to quantify the efficiency of the functionalization. To deactivate the remaining o-acylisourea ester groups on MNPs, MNPs were resuspended in 250 µL of 1 M ethanolamine and 0.1 M borate (pH 8.5) and agitated for 15 min at room temperature. The supernatant was removed, and the functionalized MNPs were finally resuspended in a 1 mL 1× Denhardt solution (1.8 × 10^8^ MNPs·µL^−1^) and stored at 4 °C.

To assess functionalization efficiency, the percentage of protein immobilized on the MNPs was determined by absorbance spectrophotometry at 280 nm using a Nanodrop TM 2000 (ThermoFisher Scientific, Waltham, MA, USA). The protein concentration in the supernatant after protein binding on the MNPs was compared to the initial protein concentration in the solution. The efficiency of protein immobilization is 44 ± 4% for streptavidin and 87 ± 1% for ovalbumin. As a consequence, 1 µL of the MNP suspension (1.8 × 10^8^ MNPs) contained 0.8 pmol of streptavidin (2700 ± 270 streptavidin molecules per MNP) or 1.9 pmol of ovalbumin (6500 ± 650 ovalbumin molecules per MNP).

### 2.2. Nucleic Acid Amplification

#### 2.2.1. Quantitative Polymerase Chain Reaction (qPCR)

The SSOAdvanced Universal SYBR^®^ Green Supermix (1725271) solution was purchased from Bio-Rad (Hercules, CA, USA). The primers were synthesized by Eurofins Genomics (Table 1). The target sequence, consisting of 461 base pairs, is localized on the pQE30 plasmid described in [15].

Quantitative PCR reactions were performed in a 20 μL reaction mixture containing 1 μL of pQE30 plasmid DNA (1 ng), 12.5 μL of Bio-Rad Super SSOAdvanced Universal SYBR Green Supermix, 1.6 μL of 0.5 µM 5′-P or 5′-P Biotin primers (0.8 pmoles), 1.6 μL of 0.5 µM 3′-C CY5 primer (0.8 pmoles), and 3.3 μL of deionized water using a Bio-Rad CFX96 Real-Time System. The expected amount of PCR product is thus limited to 0.8 pmoles by the primer concentration. The cycling parameters for the PCR protocol were as follows: pre-denaturation step at 95 °C for 2 min, followed by 25 cycles consisting of denaturation at 94 °C for 30 s, annealing at 52 °C for 1 min, and extension at 72 °C for 30 s. SYBR Green fluorescence was measured at the end of the extension phase. At the end of the qPCR procedure, the melting temperature was measured to check for the appearance of a single amplicon at 80 ± 0.5 °C. Negative controls without DNA template were included in each quantitative PCR run.

#### 2.2.2. Quantitative Loop-Mediated Isothermal Amplification (LAMP)

A WarmStart^®^ LAMP Kit (DNA and RNA) (E1700L) solution containing a LAMP Fluorescent Dye and the plasmid target pUC19 (N3041L) were provided by New England Biolabs. The six primers were designed with the New England Biolabs LAMP Primer Design Tool and synthesized by Eurofins Genomics (Table 2).

Quantitative LAMP reactions were performed in a 25 μL reaction mixture containing 1 μL of pUC19 plasmid DNA (1 ng), 12.5 μL of WarmStart^®^ LAMP Kit (DNA and RNA), 0.5 µL of LAMP Fluorescent Dye, 8.5 µL of deionized water, and 2.5 μL LAMP primer mix, using a Bio-Rad CFX96 Real-Time System. The four LAMP products and the primer concentrations in the LAMP primer mix are listed in Table 3.

The LAMP reactions were performed at 65 °C for 30 min and fluorescence was measured every minute. Negative controls without DNA templates were included in each quantitative LAMP run.

#### 2.2.3. Quantification of PCR- or LAMP-Amplified DNA

The amount of PCR- or LAMP-amplified DNA at the end of the experiment was quantified on a 1% agarose gel after electrophoresis at 100 Volts for 45 min using a Chemidoc^®^ instrument (Bio-Rad) and SYBR Safe DNA stain (S33102, Thermofisher Scientific), according to the recommendation of the supplier. Ten microliters of each amplification reaction was mixed with 2 µL of Thermo Scientific DNA Gel Loading buffer (R0611, Thermofisher Scientific). The ready-to-use MassRuler™ DNA Ladder Mix (R0611, Thermofisher Scientific) was used as a molecular weight marker. Using 1 ng of plasmid as a starting material and the protocol described above, the average amount of PCR product obtained was 168 ± 5 ng, corresponding to 0.82 pmol of a 461 bp fragment. Using 1 ng of plasmid as starting material and the protocol described above, the average amount of LAMP product obtained was 740 ± 25 ng for both FIP CY5 and LF CY5.

### 2.3. Quantitation of PCR and LAMP Products Binding on Streptavidin-Coated Magnetic Nanoparticles

DNA was amplified by PCR or LAMP from 1 ng of pQE30 or pUC19 plasmid, respectively using the primers described before, and 168 ± 5 ng (PCR) and 740 ± 25 ng (LAMP) of biotinylated or non-biotinylated fluorescent DNA was obtained. Eppendorf tubes were prepared with different amounts of streptavidin- or ovalbumin-coated MNPs, as indicated. MNPs were magnetically trapped to remove the storage buffer. The amplified DNA was added and incubated for 5 min with agitation with different amounts of streptavidin or ovalbumin-coated MNPs. MNPs were collected magnetically, and the supernatant was removed. MNPs were washed with 200 µL PBS and resuspended in 100 µL PBS. The cyanine 5 fluorescence of the resuspended MNPs and of the supernatants was measured using a microplate spectrofluorometer (Tecan Infinite^®^ m1000, λ_ex_ = 651 nm, λ_em_ = 670 nm). Results were compared to the cyanine 5 fluorescence of the initial amplified DNA sample, diluted in 100 µL, to check that cyanine 5 fluorescence was fully recovered.

The data were analyzed with a model taking into account non-specific binding (linear contribution) and saturable binding of biotinylated fluorescent molecules on streptavidin MNPs (binding capacity B_max_ and apparent affinity K). The amount of MNPs required to bind 67% of the biotinylated fluorescent molecules was equal to 2*K.

### 2.4. Detection of Amplified DNA Samples with the MagIA Analyzer

For these experiments, the same consumables (18 chamber cartridges) and equipment (MagActivator, MagIA analyzer) used to capture and detect immune complexes [13] were used to capture and detect fluorescently labeled biotinylated DNA.

As described before, 168 ng of biotinylated or non-biotinylated fluorescent PCR amplicons and 740 ng of biotinylated or non-biotinylated fluorescent LAMP amplicons were prepared. A range of amplification products (from 0 to 168 ng for PCR products and from 0 to 740 ng for LAMP products) was prepared by diluting the amplified DNA in PBS.

Five microliters of streptavidin-coated MNPs (6 × 10^9^ MNPs) and twenty microliters of diluted PCR or LAMP product containing the indicated amount of DNA were mixed in a microtube. The mixture was incubated at room temperature for 5 min. Five microliters of the mixture was then injected into a single well of an 18-well cartridge. Triplicates were prepared. Once all the wells were filled, the entire cartridge was placed on the MagActivator for 1 min to activate the capture of magnetic nanoparticles. The activated cartridge was then inserted into the analyzer. Ovalbumin-coated MNPs were used in the same way as the negative control.

### 2.5. MagIA Analyzer

Cartridges and MagActivator are described in [13]. The device used to acquire MLF signals is also described in [13] as an MLFIA analyzer. Briefly, it consists of a miniaturized epifluorescence microscope allowing far-red fluorescence imaging of cyanine 5. A mechanical module allows one to automatically displace the cartridge under the optical axis. Three pictures of each well are acquired at three different exposure times. The pictures having the largest grey level dynamic range are selected and processed as described below.

### 2.6. Image Processing

The acquired pictures were processed as described in [13]. Briefly, the images were automatically processed to filter artifacts (stains, bubbles, etc.). The orientation of the lines was adjusted along the vertical axis. Finally, the fluorescence profile was integrated along the vertical axis (micro-magnet junctions), and the specific signal was calculated with respect to the background signal (between the micro-magnet junctions).

### 2.7. DNA Amplification Kinetics

The limit of detection (LOD) is defined as the minimum amount of DNA producing a signal higher than the statistically significant minimum detectable signal (MDS):

(1) $$\mathrm{MDS}=3\sigma_{0}$$

(2) $$\mathrm{LOD}=\frac{3\sigma_{0}}{\alpha }$$

where *σ*_0_ values are the average and standard deviation of the signal measured at t = 0 or in the absence of DNA template and α is the initial slope of the calibration curve (signal vs. initial amount of DNA).

## 3. Results

### 3.1. Preparation and Characterization of Amplified DNA Samples

To assess the ability of the previously developed MLFIA technology to detect nucleic acid amplification, amplified DNA was prepared and characterized using two methods, PCR and LAMP. Biotinylated primers were used to ensure capture by streptavidin-coated MNPs and CY5-modified primers for fluorescence detection in the analyzer.

**Figure 2:**
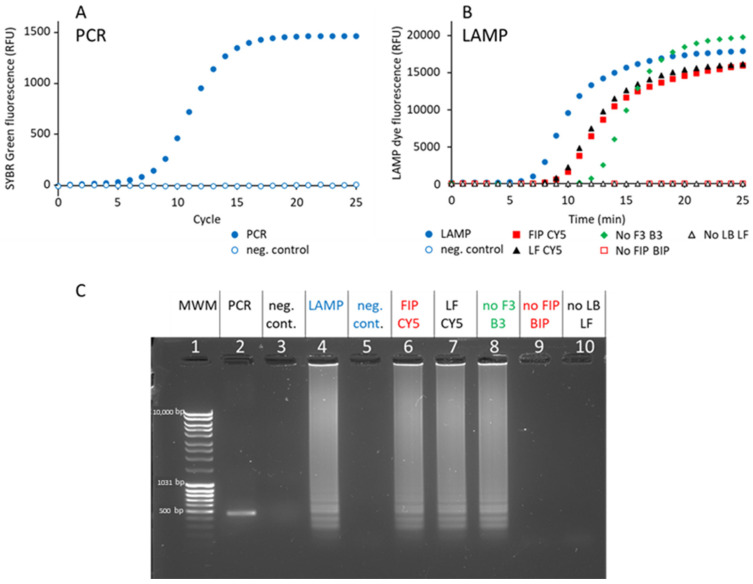
Characterization of amplified DNA samples. (**A**) qPCR amplification kinetics. The SYBR Green fluorescent signal is plotted as a function of the cycle number. PCR: PCR positive control. Negative control: reaction without template DNA. Data are the average of three replicates. The standard deviation is smaller than the size of the symbols. The coefficient of variation of the specific signal is 1%. (**B**) LAMP amplification kinetics. The LAMP fluorescent dye signal is plotted as a function of time. LAMP: LAMP reaction with F3, B3, FIP, BIP, LF, and LB primers without modification. Negative control: same reaction without template DNA. FIP CY5: LAMP reaction with fluorescent FIP CY5 and biotinylated LF primer FIP CY5 LAMP kinetics. LF CY5: LF CY5 LAMP kinetics. No F3 B3: LAMP reaction without primers F3 and B3. No FIP BIP: LAMP reaction without primers FIP and BIP. No LF LB: LAMP reaction without primers LF and LB. Data are the average of three replicates. The coefficients of variation of the LAMP, FIP CY5, LF CY5, and No F3 B3 kinetics are 5%, 6%, 7%, and 13%, respectively. (**C**) Agarose gel (1%) electrophoresis analysis of the amplicons generated. Lane 1: Molecular weight marker (MWM). Lane 2: 461 bp PCR amplicon synthesized using biotinylated 5′-P Biotin and 3′-P cyanine 5. Lane 3: negative PCR control without template DNA. Lane 4: LAMP amplicons obtained with F3, B3, FIP, BIP, LF, and LB primers without modification. Lane 5: negative LAMP control without template DNA. Lane 6: FIP CY5 LAMP product. Lane 7: LF CY5 product. Lane 8: LAMP amplicons generated without primers F3 and B3. Lane 9: LAMP reaction without primers FIP and BIP. Lane 10: LAMP reaction without primers LF and LB. The gel shown is representative of at least 3 independent experiments.

First, a 461 bp DNA fragment was amplified by PCR from a pQE30 plasmid using biotinylated and fluorescent primers. Using 1 ng plasmid DNA as a template, 15 cycles were necessary to attain saturation (Figure 2A). Agarose gel analysis confirmed that a single band was amplified at the expected molecular size (Figure 2C, lane 2). The amount of DNA obtained corresponds to the quantity of primer added, showing that the PCR reaction is limited by primer availability.

Second, a 161 bp DNA fragment was amplified by LAMP from a pUC19 plasmid, using six primers (F3, B3, FIP, BIP, LF and LB, Table 2), as defined in [16]. Full amplification was attained after 20 min (Figure 2B). Agarose gel analysis confirmed that the amplified material consists of many bands (Figure 2C, lane 4), as expected from LAMP [10]. The smallest fragment has an apparent size of 170 bp, which matches the expected size of the 161 bp fragment. Some LAMP amplification structures remain trapped in the well during agarose gel electrophoresis due to their weight and complex secondary structures (Figure 2C, lanes 4, 6, 7, and 8). Unlike linear PCR amplicons, LAMP products form high-molecular-weight concatemers, loops, and branched structures, which hinder migration through our 1% agarose gel [17].

Primers F3 and B3 are dispensable, but their presence accelerates amplification (Figure 2B). In contrast, the primer pairs FIP/BIP and LB/LF are essential, since, in their absence, no amplification is detected after 1h (Figure 2C, lanes 9 and 10). In order to detect LAMP-amplified samples, biotinylated and cyanine-5-modified FIP and LF primers were used, according to the recommendations of [18]. We tested two configurations: in the FIP CY5 sample, LF was biotinylated and FIP was modified with CY5, and in the LF CY5 sample, FIP was biotinylated (Table 3). As controls, samples were prepared without biotinylated primers (FIP CY5 without biotin and LF CY5 without biotin). The amplification kinetics of these four samples was not different from the one obtained with the non-modified primers (Figure 2B). Similarly, no difference was observed on agarose gel, demonstrating that the primer 5′ modification does not interfere with LAMP amplification (Figure 2C, lanes 6 and 7).

### 3.2. Binding Capacity of Streptavidin-Coated Magnetic Nanoparticles

**Figure 3:**
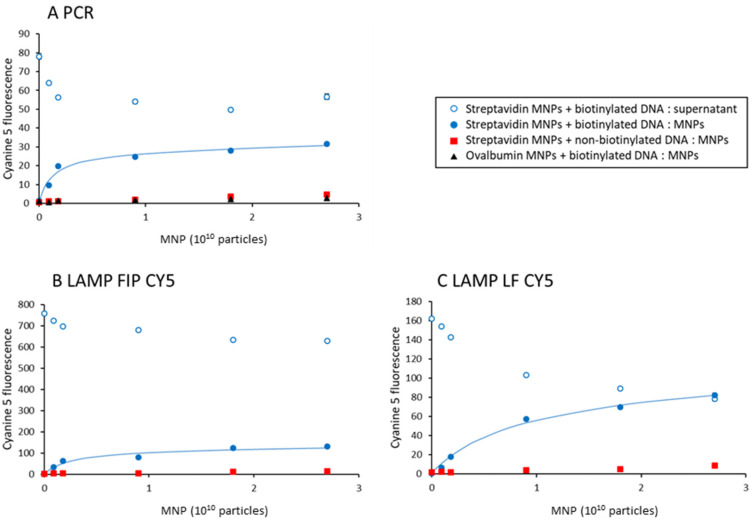
Capture of amplified DNA samples on functionalized MNPs. Biotinylated fluorescent amplified DNA samples were incubated with increasing amounts of streptavidin-coated MNPs. The supernatant was separated from MNPs by centrifugation and the cyanine 5 fluorescence contained in the supernatant (open circles) or in the MNPs (closed circles) was quantified and plotted as a function of the number of MNPs in the assay. As a control, reactions were carried out with non-biotinylated amplified DNA (squares) or ovalbumin-coated MNPs (triangles). (**A**) PCR-amplified samples. (**B**) LAMP FIP CY5-amplified samples. (**C**) LAMP LF CY5-amplified samples. The binding of biotinylated fluorescent molecules on streptavidin-coated MNPs was fitted to extract the binding capacity B_max_ and the apparent affinity K. The binding capacity is 27, 121, and 98 fluorescence units for the PCR, FIP CY5, and LF CY5 samples, respectively. The apparent affinity is 1.3 × 10^9^, 2.6 × 10^9^, and 8.7 × 10^9^ MNPs for PCR, LAMP FIP CY5, and LAMP LF CY5, respectively. The results are the average of 3 samples. The standard deviation is smaller than the symbol size.

Streptavidin- or ovalbumin-coated magnetic nanoparticles were prepared as described in Materials and Methods. In order to characterize their binding capacity, biotinylated or non-biotinylated cyanine-5-fluorescent DNA molecules were prepared by PCR and LAMP. The fluorescence in these samples (78, 161, and 758 fluorescence units) for PCR, LF CY5, and FIP CY5 LAMP samples depends on the amount of the cyanine 5 primer, i.e., 0.8, 1.3, and 5.1 pmol, respectively, and is used as a reference (100%). Figure 3 displays the amount of cyanine 5 fluorescence captured on the MNPs after 5 min incubation, as a function of the total number of MNPs in a volume of 100 µL. Five minutes was chosen because this is comparable to the incubation time in MLFIA technology [19].

In Figure 3A, 41 ± 1% of the fluorescent PCR product can be specifically captured on streptavidin-coated MNPs (binding capacity divided by the total of 78 fluorescence units). The amount of non-specifically bound DNA is less than 5%, as determined with ovalbumin-coated MNPs. Similarly, non-biotinylated DNA does not bind appreciably to streptavidin-coated MNPs. The incomplete capture of the fluorescent PCR product indicates that the primers are not all modified in 5′. Subtracting non-specific binding, 2.6 × 10^9^ MNPs are required to capture 67% of the amplified PCR product (0.52 pmol) corresponding to 12 pmol of immobilized streptavidin. The binding capacity of the MNPs is therefore 0.23 pmol·µL^−1^ (110 biotinylated DNA molecules per MNP).

As shown in Figure 2C lane 4, LAMP amplification results in many different amplified molecules, and only some of them can be labeled with both biotinylated and fluorescence primers [18]. We therefore compared two samples, one obtained with cyanine 5 labeling of the primer FIP and biotinylated primer LF (FIP CY5, Figure 3B), the other obtained with cyanine 5 labeling of the primer LF and biotinylated primer FIP (LF CY5, Figure 3C). The amount of cyanine-5-labeled DNA specifically captured on streptavidin-coated MNPs is strongly reduced for FIP CY5 samples compared to LF CY5 ones (15 ± 1% versus 46 ± 2%, respectively). The amount of MNPs required to capture 67% of the amplified product is also reduced in the same proportion (5.2 × 10^9^ and 1.7 × 10^10^ for FIP CY5 and LF CY5, respectively). This indicates that biotinylated primers are present in a fraction of fluorescent amplified DNA.

Finally, it should be noted that the total amount of cyanine 5 fluorescent signal that can be captured on MNPs is significantly larger for LAMP-amplified DNA than for PCR amplified DNA: 116 and 74 fluorescence units for FIP CY5 and LF CY5 LAMP products versus 27 for PCR products.

### 3.3. Detection of Amplified DNA Samples with the MLF Technology

**Figure 4:**
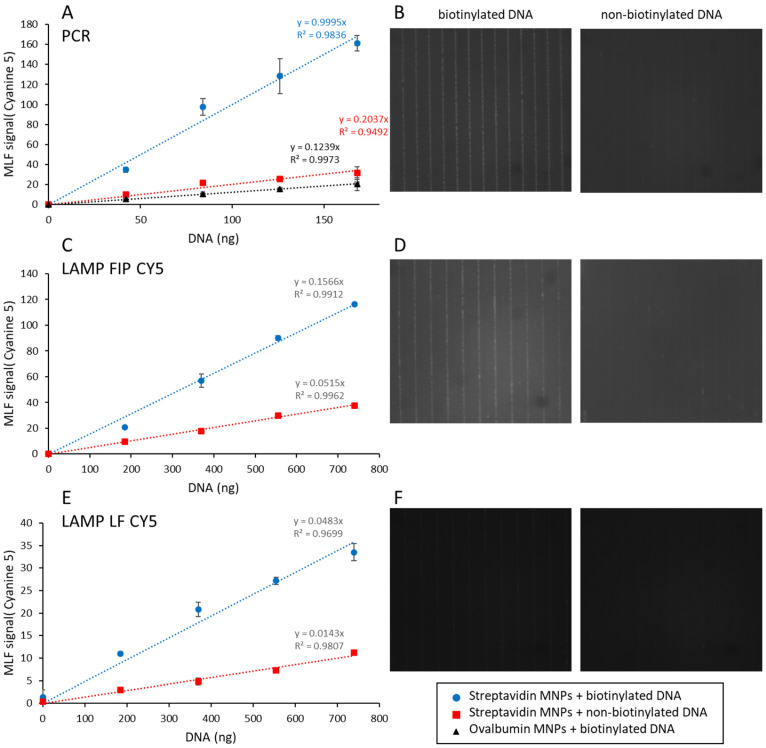
Magnetically localized fluorescent signals as a function of amplified DNA concentration. One hundred sixty-eight or seven hundred fifty nanograms of biotinylated fluorescent DNA was generated by PCR (**A**,**B**) or LAMP using fluorescent FIP (**C**,**D**) or LF primers (**E**,**F**). Amplicons were captured on streptavidin-coated MNPs (blue circles in **A**,**C**,**E**; left picture in **B**,**D**,**F**) or ovalbumin-coated MNPs (black triangles in **A**) and analyzed with a MagIA analyzer. As controls, non-biotinylated PCR or LAMP products were generated and captured on streptavidin-coated MNPs (red squares in **A**,**C**,**E**; right picture in **B**,**D**,**F**). (**A**,**C**,**E**) Magnetically localized fluorescent (MLF) signals as a function of amplified DNA concentration. The MLF signal obtained in the absence of amplified DNA was subtracted from all measurements. The straight lines are a linear regression through the origin. The results are the average of 6 samples. (**B**,**D**,**F**) Representative cyanine 5 fluorescence images of biotinylated (**left**) or non-biontinylated (**right**) amplicons captured on micromagnets by streptavidin-coated MNPs. Images in **B** are acquired with 700 ms exposure time, and images in **D** and **F** are acquired with 50 ms exposure time.

Different amounts of PCR- or LAMP-amplified biotinylated fluorescent DNA were added to a constant amount (10^9^ MNPs) of streptavidin-coated MNPs (Figure 4).

This amount of MNPs, insufficient to capture all DNA, is the same as the amount used in [13]. After 5 min incubation, 5 µL of the reaction mixture was deposited in a cartridge chamber integrating a micro-magnet array consisting of stripes of alternating magnetization. The cartridge was then magnetized with the MagActivator to launch the capture of MNPs at the junctions of the micro-magnet arrays. The cartridge was then inserted in the MagIA analyzer where the fluorescence associated with the MNPs was quantified and compared to the fluorescence of the background, resulting in an MLF signal [13]. The value of the MLF signal obtained with MNPs in the absence of amplified DNA (0.5 ± 0.2) was subtracted from all measurements. The values of this zero are the fluorescence signal of the MNPs in PBS. The minimum detectable signal is about 1 and the maximum signal is higher than 160.

Samples prepared without biotinylated primers indicate a contribution of non-specific DNA binding to the MLF signal. This contribution is higher for LAMP-amplified products than for PCR ones (Figure 4A,C,E).

### 3.4. Monitoring DNA Amplification by Magnetically Localized Fluorescence

In the previous experiments, the ratio of the amount of cyanine 5 fluorescence to the number of MNPs varied as the total amount of amplified DNA varied. In order to better characterize the detection limit of MLF, we ran DNA amplification kinetics and compared the cyanine 5 MLF signals measured in the MagIA analyzer to the standard fluorescent signals elicited by intercalating dyes recorded on a qPCR apparatus (Bio-Rad CFX96). For this purpose, 1 ng of DNA was amplified by PCR or LAMP, in a 20 µL volume, using the primer sets defined before. After a defined number of cycles (PCR), or at defined times (LAMP), the fluorescence of PCR or LAMP samples was measured in the Bio-Rad CFX96 Real-Time System, using SYBR Green or LAMP fluorescent dye, respectively. The samples were then mixed with 5 µL of streptavidin-coated MNPs (6 × 10^9^ MNPs) and incubated for 5 min. Three 5 µL aliquots were analyzed in the analyzer as previously described.

The values of the MLF signals obtained in the absence of DNA amplification by PCR is (19 ± 1), LAMP FIP CY5 (36 ± 2), and LF CY5 (9 ± 1) were subtracted from all measurements. These values represent the fluorescence signal of MNPs and the amplification mix without amplification.

**Figure 5:**
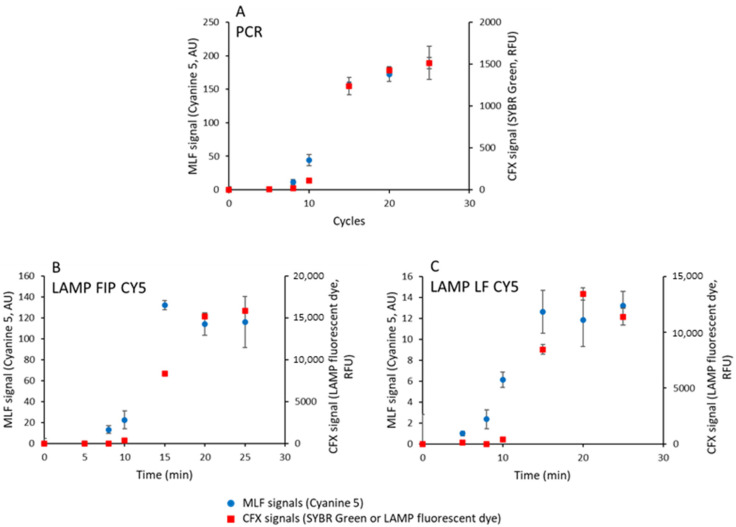
Compared sensitivity of magnetically localized fluorescent technology and DNA intercalation for the detection of amplified DNA products. One nanogram of plasmid DNA was amplified by PCR (**A**) or by LAMP using an FIP CY5 (**B**) or LF CY5 (**C**) set of primers incorporating biotinylated and cyanine 5-modified primers in the presence of SYBR Green (PCR) or LAMP fluorescent dye (LAMP). The kinetics were monitored in parallel using magnetically localized fluorescent signals (MLF, circles) and DNA-intercalated fluorescent dyes (CFX, squares). The results are the average of 8 samples, except LAMP FIP CY5 (16 samples).

In Figure 5A–C, the kinetics of the intercalating dye signal (CFX signal) are compared to the kinetics of cyanine 5 MLF signals for PCR, LAMP FIP CY5, and LF CY5 samples, respectively.

The maximum scales of MLF and CFX signals were adjusted for the sake of comparison. For PCR, the kinetics of intercalating dye CFX and cyanine 5 MLF signals are identical, within the error bars. In contrast, MLF signals an increase before CFX signals for both LAMP amplifications (Figure 5B,C).

Concerning LAMP, for LF CY5 samples, MLF and CFX signals have comparable kinetics, indicating that the MLF signal is proportional to the DNA content of the sample. Using intercalating fluorescent dye (CFX recordings), the LF CY5 and FIP CY5 LAMP kinetics are indistinguishable. However, for FIP CY5 LAMP samples, the maximum MLF signal is attained at intermediate times (15 min) and not a final time, suggesting that a reaction intermediate contributes to the MLF signal and not simply the total amplified DNA.

### 3.5. Quantifying Initial DNA by Magnetically Localized Fluorescence

**Figure 6:**
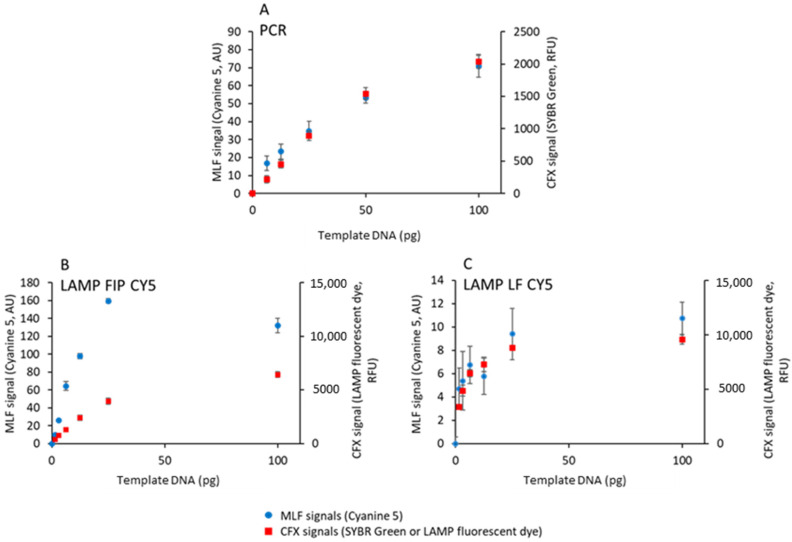
Quantitative detection of template plasmid DNA. A total of 15 cycles of PCR (**A**) or 15 min of LAMP (**B**,**C**) were performed with the indicated amounts of template plasmid DNA in the presence of SYBR Green (**A**) or LAMP fluorescent dye (**B**,**C**) using biotinylated and cyanine-5-labeled primers. The samples were then processed to record MLF (circles) and CFX signals (squares). The results are the average of 8 samples.

To compare the sensitivity of the two techniques for the detection of PCR and LAMP products, MLF and CFX signals were acquired after 15 cycles or 15 min using various amounts of template DNA plasmid and biotinylated and cyanine-5-labeled primers, in the presence of SYBR Green or LAMP fluorescent dye (Figure 6).

All techniques allow quantifying the initial DNA concentration up to 25–50 ng and saturating above such a concentration. The MDS defined by Equation (1) was used to compute the limit of detection (LOD) for the MLF and CFX signals according to Equation (2). LOD values in Table 4 show that the commercial quantitative PCR instrument is two orders of magnitude more sensitive than the MagIA analyzer for both PCR and LAMP detection. This difference is explained by the very large dynamics of quantitative PCR compared to MLF technology.

## 4. Discussion

**Figure 7:**
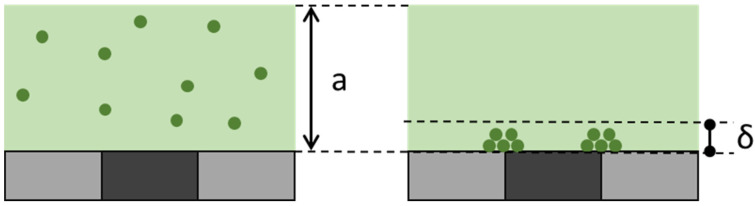
Concentration of fluorescent signal at the level of the micromagnets. The **left** and **right** panels represent the fluorescence distribution before and after the capture of the MNPs on the macromagnets. Part of the fluorescence is in solution (unbound signal, light green) and part of the fluorescence is specifically captured on the MNPs (dark green on the beads). During imaging, the limited depth of focus of the microscope captures a limited volume close to the micromagnets. This volume contains all the MNPs but only a fraction of the unbound signal.

As demonstrated above, MLFMA allows the detection of cyanine-5-labeled biotinylated amplified DNA molecules, obtained by either PCR or LAMP. MLF signals measure the fluorescence associated with the MNPs captured on the micro-magnet lines in the detection chamber. The specific signal therefore corresponds to bound cyanine 5 molecules, whereas the non-specific signal is due to free cyanine 5 ones. Local MNPs captured on micro-magnets separate free and bound fluorophores in the capture zone, as explained by Fratzl et al. [19]. This differential mode of detection is quite sensitive, because the focus depth of the microscope (δ ≈ 10 µm) is smaller than the extent of the capture zone (a ≈ 100 µm), as shown in Figure 7.

This explains why MLF signals have a good signal-to-noise ratio, although the amount of free fluorophores largely exceeds the amount of bound fluorophores. A comparable effect was reported by Fu et al. [20].

Compared to standard intercalating fluorophores, the total number of fluorophores per DNA molecule is lower in MLFMA (one per amplified PCR fragment) than in qPCR machines (about 35 SYBR Green molecules per base pairs [21]). This is compensated by the five-fold higher extinction coefficient of cyanine 5 (2.5 × 10^5^ M^−1^·cm^−1^) compared to SYBR Green (5.7 × 10^4^ M^−1^·cm^−1^) [22]. The fluorescence intensity of the detection could be further increased using a cyanine 5-dATP nucleotide analogue to directly label DNA molecules [23].

The limited binding capacity of streptavidin-coated MNPs appears as the Achilles heel of MLF. First, the streptavidin-coated MNPs have a limited binding capacity: 110 biotinylated PCR molecules for 2700 streptavidin per bead. This 4% binding capacity limits the maximum signal that MLFMA can detect and therefore the dynamics of the detection. This low value is likely explained by electrostatic repulsion between bound and incoming DNA molecules. In PBS, the Debye length is 0.72 nm, which prevents DNA molecules from coming closer than a few nanometers. The distance between streptavidin molecules on streptavidin-coated MNPs is indeed smaller (17 nm) than the size of PCR products (157 nm). The 4% coverage indicates that the average distance between captured PCR products is 85 nm, which better matches the size of DNA molecules and limits electrostatic repulsion between adjacent DNA molecules. Functionalizing the magnetic MNPs with streptavidin-dextran conjugates could relieve this limitation and greatly increase the amount of fluorescent DNA captured per MNPs [24].

With the MLFMA technology, we were able to detect LAMP-amplified DNA both at early times (5 min) and at the endpoint (20 min). In LAMP detection, we face the same limitation due to the binding capacity of MNPs. This constraint may be even more significant because LAMP products are larger, leading not only to electrostatic repulsion but also to steric hindrance. As a result, the capture of amplified products by MNPs is limited not only by the distance between DNA molecules due to electrostatic forces [25] but also by the spatial crowding of the larger products (Figure 2C), further reducing the overall potential detection efficiency.

The primer combination used in the LF CY5 samples allowed for defining an optimum time point for detection at 15 min. The MLFMA technique is therefore suitable for the quantitative detection of LAMP-amplified DNA products in portable instruments.

Improving the signal-to-noise ratio is crucial for enhancing the sensitivity of our detection system. A higher signal-to-noise ratio means that the true positive signals are more distinguishable from background noise, leading to more accurate results. To achieve this, it is essential to

(1) Reduce background noise: By reducing the variability issues inherent to the MagIA platform described in [13] (focus, dust, capture, etc.), the variability of the negative samples and thus the LOD would be reduced. This enables the system to detect smaller initial quantities of DNA, increasing its sensitivity and effectiveness in identifying low-concentration targets.
(2) Improve the grafting efficiency to capture more DNA, leading to more consistent signal generation. Additionally, optimizing the grafting process will increase the dynamic range of detection.

These improvements would enhance the accuracy and reliability at low and high initial DNA levels.

## 5. Conclusions

Our current device demonstrates significant potential to replace traditional methods for confirming amplification results in research laboratories, particularly by eliminating the need for agarose gels. Used in combination with existing compact PCR devices, it could serve as a cost-effective alternative to expensive real-time thermal cyclers. Its precision and simplicity allow for effective integration into laboratory workflows, optimizing the time and resources required for diagnostic tests.

The compact and user-friendly nature of the device not only facilitates the execution of immunoassays but also enables the accurate and quantitative detection of amplified DNA with greater ease. Its versatility and adaptability to detect different targets (proteins and nucleic acids) and accessibility make it an ideal tool for a wide range of in vitro applications, such as human and veterinary [26] infectious disease diagnostics, where immunoassays and molecular tests are complementary [27]. It is particularly suitable for small laboratories with limited resources and low sample throughput.

The sensitivity of MLFMA is not yet equivalent to that of a commercial quantitative PCR apparatus. However, this device represents a crucial step toward the future development of point-of-care diagnostic tools. By integrating the different steps of the MLFMA, it will be possible to develop an even more portable and user-friendly solution, facilitating rapid and reliable diagnostics even in resource-limited settings. These technological advancements promise to transform diagnostic practices, making tests more accessible and enabling quicker and more efficient disease management. This work highlights the capability to detect and quantify amplified nucleic acids by either PCR or LAMP using a device originally designed for immunoassays, demonstrating the platform’s ability for combined diagnostics and its promise for broader applications in diagnostics.

## Figures and Tables

**Table 1:** Sequence and 5′ modification of PCR primers.

Primer	5′ Modification	DNA Sequence
5′-P Biotin	Biotin	TTTTTTCGCATCACCATCACCAT
5′-P	None	TTTTTTCGCATCACCATCACCAT
3′-P CY5	Cyanine 5	TTTTTTGCTTGGCTGCAGGTCGACCCGGG

**Table 2:** Sequence and 5′-modification of LAMP primers.

Primer	5′ Modification	DNA Sequence
F3	None	GCTGCAAGGCGATTAAGTTG
B3	None	CACCCCAGGCTTTACACTT
FIP CY5	Cyanine 5	TAGAGGATCCCCGGGTACCGACCAGGGTTTTCCCAGTCAC
FIP Biotin	Biotin	TAGAGGATCCCCGGGTACCGACCAGGGTTTTCCCAGTCAC
BIP	None	CAGGCATGCAAGCTTGGCGTATTGTGTGGAATTGTGAGCGG
LF CY5	Cyanine 5	TGGCCGTCGTTTTACAACGTC
LF Biotin	Biotin	TGGCCGTCGTTTTACAACGTC
LB	None	CATGGTCATAGCTGTTTCCTGTGT

**Table 3:** LAMP products obtained with different primer sets.

LAMP Product Name	Primers	Primer Concentrations
FIP CY5	F3/B3	0.4 µM
	FIP CY5/BIP	3.2 µM
	LF Biotin/LB	0.8 µM
LF CY5	F3/B3	0.4 µM
	FIP Biotin/BIP	3.2 µM
	LF CY5/LB	0.8 µM
FIP CY5 without biotin	F3/B3	0.4 µM
	FIP CY5/BIP	3.2 µM
	LF/LB	0.8 µM
LF CY5 without biotin	F3/B3	0.4 µM
	FIP/BIP	3.2 µM
	LF CY5/LB	0.8 µM

**Table 4:** MDS and LOD of MLF and CFX signals.

Method	Instrument	MDS (A.U.)	Initial DNA Limit of Detection (pg)
PCR	CFX quantitative PCR	0.7	0.02
	MagIA analyzer	4.5	3.80
FIP CY5 LAMP	CFX quantitative LAMP	3.1	0.02
	MagIA analyzer	5.5	0.8
LF CY5 LAMP	CFX quantitative LAMP	9.5	0.01
	MagIA analyzer	1.7	1.3

## Data Availability

The raw data supporting the conclusions of this article will be made available by the authors on request.

## Abbreviations

The following abbreviations are used in this manuscript:

CY5	Cyanine 5
EDC	1-ethyl-3-(3-dimethylaminopropyl) carbodiimide
LAMP	Loop-mediated isothermal Amplification
LFA	Lateral flow assay
LOD	Limit of detection
MDS	Minimum detectable system
MNP	Magnetic nanoparticle
MLF	Magnetically localized fluorescence
MLFIA	Magnetically localized fluorescence immunoassay
MLFMA	Magnetically localized fluorescence molecular assay
PCR	Polymerase chain reaction

## References

[1] Mullis K., Faloona F., Scharf S., Saiki R., Horn G., Erlich H. (1992). Specific enzymatic amplification of DNA in vitro: The polymerase chain reaction. 1986. Biotechnology.

[2] Witt N., Rodger W., Vandesompele J., Benes V. (2009). An assessment of air as a source of DNA contamination encountered when performing PCR. J. Biomol. Tech..

[3] Wang R., Carter J., Lench N. (2013). Evaluation of real-time quantitative PCR as a standard cytogenetic diagnostic tool for confirmation of microarray (aCGH) results and determination of inheritance. Genet. Test. Mol. Biomark..

[4] Bustin S.A., Nolan T. (2020). RT-QPCR testing of SARS-COV-2: A primer. Int. J. Mol. Sci..

[5] Garcia-Diaz M., Bebenek K. (2007). Multiple functions of DNA polymerases. CRC Crit. Rev. Plant Sci..

[6] Cobb B., Simon C.O., Stramer S.L., Body B., Mitchell P.S., Reisch N., Stevens W., Carmona S., Katz L., Will S. (2017). The cobas^®^ 6800/8800 System: A new era of automation in molecular diagnostics. Expert. Rev. Mol. Diagn..

[7] Babady N.E. (2013). The FilmArray^®^ respiratory panel: An automated, broadly multiplexed molecular test for the rapid and accurate detection of respiratory pathogens. Expert. Rev. Mol. Diagn..

[8] Gaydos C.A., Van Der Pol B., Jett-Goheen M., Barnes M., Quinn N., Clark C., Daniel G.E., Dixon P.B., Hook E.W. (2013). Performance of the cepheid CT/NG Xpert rapid PCR test for detection of Chlamydia trachomatis and Neisseria gonorrhoeae. J. Clin. Microbiol..

[9] Moeller M.E., Engsig F.N., Bade M., Fock J., Pah P., Soerensen A.L., Bang D., Donolato M., Benfield T. (2022). Rapid Quantitative Point-Of-Care Diagnostic Test for Post COVID-19 Vaccination Antibody Monitoring. Microbiol. Spectr..

[10] Mori Y., Kitao M., Tomita N., Notomi T. (2004). Real-time turbidimetry of LAMP reaction for quantifying template DNA. J. Biochem. Biophys. Methods.

[11] Cuadros J., Martin Ramírez A., González I.J., Ding X.C., Perez Tanoira R., Rojo-Marcos G., Gómez-Herruz P., Rubio J.M. (2017). LAMP kit for diagnosis of non-falciparum malaria in Plasmodium ovale infected patients. Malar. J..

[12] Ljolje D., Abdallah R., Lucchi N.W. (2021). Detection of malaria parasites in samples from returning US travelers using the Alethia^®^ Malaria Plus LAMP assay. BMC Res. Notes.

[13] Delshadi S., Fratzl M., Ramel O., Bigotte P., Kauffmann P., Kirk D., Masse V., Brenier-Pinchart M.P., Fricker-Hidalgo H., Pelloux H. (2023). Magnetically localized and wash-free fluorescence immunoassay (MLFIA): Proof of concept and clinical applications. Lab. Chip.

[14] Fratzl M., Bigotte P., Gorbenkov R., Goubet G., Halfon P., Kauffmann P., Kirk D., Masse V., Payet-Burin X., Ramel O. (2024). Magnetically localized and wash-free fluorescent immuno-assay: From a research platform (MLFIA) to a multiplexed POC system (MagIA). SLAS Technol..

[15] Bogdanovic A., Bennett N., Kieffer S., Louwagie M., Morio T., Garin J., Satre M., Bruckert F. (2002). Syntaxin 7, syntaxin 8, Vti1 and VAMP7 (vesicle-associated membrane protein 7) form an active SNARE complex for early macropinocytic compartment fusion in *Dictyostelium discoideum*. Biochem. J..

[16] Nagamine K., Hase T., Notomi T. (2002). Accelerated reaction by loop-mediated isothermal amplification using loop primers. Mol. Cell Probes.

[17] Notomi T., Okayama H., Masubuchi H., Yonekawa T., Watanabe K., Amino N., Hase T. (2000). Loop-mediated isothermal amplification of DNA. Nucleic Acids Res..

[18] Xing J., Yu J., Liu Y. (2020). Improvement and evaluation of loop-mediated isothermal amplification combined with chromatographic flow dipstick assays for Vibrio parahaemolyticus. J. Microbiol. Methods.

[19] Fratzl M., Delshadi S., Devillers T., Bruckert F., Cugat O., Dempsey N.M., Blaire G. (2018). Magnetophoretic induced convective capture of highly diffusive superparamagnetic nanoparticles. Soft Matter.

[20] Fu Y., Zhang J., Lakowicz J.R. (2009). Highly efficient detection of single fluorophores in blood serum samples with high autofluorescence. Photochem. Photobiol..

[21] Lucchi N.W., Gaye M., Diallo M.A., Goldman I.F., Ljolje D., Deme A.B., Badiane A., Ndiaye Y.D., Barnwell J.W., Udhayakumar V. (2016). Evaluation of the Illumigene Malaria LAMP: A Robust Molecular Diagnostic Tool for Malaria Parasites. Sci. Rep..

[22] Kolbeck P.J., Vanderlinden W., Gemmecker G., Gebhardt C., Lehmann M., Lak A., Nicolaus T., Cordes T., Lipfert J. (2021). Molecular structure, DNA binding mode, photophysical properties and recommendations for use of SYBR Gold. Nucleic Acids Res..

[23] Yu H., Chao J., Patek D., Mujumdar R., Mujumdar S., Waggoner A.S. (1994). Cyanine dye dUTP analogs for enzymatic labeling of DNA probes. Nucleic Acids Res..

[24] Morimoto J., Sarkar M., Kenrick S., Kodadek T. (2014). Dextran as a generally applicable multivalent scaffold for improving immunoglobulin-binding affinities of peptide and peptidomimetic ligands. Bioconjug Chem..

[25] Hong S.H., Seo K.H., Yoon S.H., Kim S.K., Chon J. (2023). Gold Nanoparticle and Polymerase Chain Reaction (PCR)-Based Colorimetric Assay for the Identification of Campylobacter spp. in Chicken Carcass. Food Sci. Anim. Resour..

[26] Tumino S., Tolone M., Parco A., Puleio R., Arcoleo G., Manno C., Nicholas R.A.J., Loria G.R. (2020). Validation of loop-mediated isothermal amplification (LAMP) field tool for rapid and sensitive diagnosis of contagious agalactia in small ruminants. Animals.

[27] Ling M.M., Ricks C., Lea P. (2007). Multiplexing molecular diagnostics and immunoassays using emerging microarray technologies. Expert Rev. Mol. Diagn..

